# Performance Evaluation of UOWC Systems from an Empirical Channel Model Approach for Air Bubble-Induced Scattering

**DOI:** 10.3390/s24165232

**Published:** 2024-08-13

**Authors:** Pedro Salcedo-Serrano, Rubén Boluda-Ruiz, José María Garrido-Balsells, Beatriz Castillo-Vázquez, Antonio Puerta-Notario, Antonio García-Zambrana

**Affiliations:** Telecommunication Research Institute (TELMA), Universidad de Málaga, E-29010 Málaga, Spain; rbr@ic.uma.es (R.B.-R.); jmgb@ic.uma.es (J.M.G.-B.); bcv@ic.uma.es (B.C.-V.); apn@ic.uma.es (A.P.-N.); agz@ic.uma.es (A.G.-Z.)

**Keywords:** underwater optical wireless communication, underwater free-space optical, air bubbles, scattering, turbid water, BER performance, outage probability

## Abstract

Underwater optical wireless communication (UOWC) systems provide the potential to establish secure high-data-rate communication links in underwater environments. The uniqueness of oceanic impairments, such as absorption, scattering, oceanic turbulence, and air bubbles demands accurate statistical channel models based on empirical measurements for the development of UOWC systems adapted to different types of water and link conditions. Recently, generalized Gamma and a mixture of two generalized Gamma probability density functions (PDF) were proposed to describe the statistical behavior of small and large air bubbles, respectively, when considering several levels of particle-induced scattering. In this paper, we derive novel closed-form analytic expressions to compute the bit error rate (BER) and outage performance using both proposed PDFs for various scattering conditions. Furthermore, simple asymptotic expressions are obtained to determine the diversity order of each scenario. Monte Carlo simulation results verify the obtained theoretical expressions. Our results also reveal that UOWC systems present lower BER and outage performance under more turbid water cases with respect to the tap water case due to the higher diversity order and despite the significant increases in pathloss at short link distances. Particle-induced scattering provides an inherent mechanism of turbid waters to mitigate air bubble-induced fluctuations and light blockages.

## 1. Introduction

The underwater medium presents significant challenges for wireless communication systems that aim to extend the telecommunication networks to marine environments [1,2]. Recent bandwidth-hungry applications such as high-definition and real-time video transmission or remote control of underwater vehicles require robust links with low delay latency. At the same time, some of these applications require high transmission security due to the sensitivity of the information. In this context, underwater optical wireless communication (UOWC) systems offer a promising alternative to acoustic wireless communication systems, especially over short and moderate link distances [3]. However, the underwater optical channel presents physical challenges that can degrade the performance of UOWC systems that are not adequately designed for underwater-specific conditions. Traditional theoretical models, often based on Monte Carlo simulations, are limited by assumptions such as idealizing scattering particles as spherical or treating air bubbles under specific conditions [4]. These assumptions may not accurately reflect the true complexity of the marine environment. Some of these assumptions are related to the absorption and scattering effect, oceanic turbulence, and the presence of air bubbles. Each of these effects exhibits different characteristics and affects the performance of the UOWC system in different ways. However, the empirical approach provides a more accurate and realistic depiction of the underwater optical channel, integrating realistic environmental conditions rather than relying on simplified theoretical assumptions. Therefore, reliable UOWC system designs for specific oceanic environments require a performance analysis based on realistic and experimental underwater channel models.

Given the difficulty of conducting experiments in the open ocean, underwater optical channel models are typically developed in laboratory water tanks under controlled conditions. In this respect, several studies have focused on the experimental modeling of UOWC channels under different phenomena, such as random temperature and salinity variations or air bubbles [5,6,7,8,9,10,11] (and references therein). Numerous works have demonstrated the importance of these models by obtaining new closed-form expressions for evaluating the UOWC system performance under these experimental channel models. The vast majority of these works have concentrated on analyzing UOWC systems under oceanic turbulence by means of probability density functions (PDF) such as the Weibull distribution [12,13], the Exponential-Generalized Gamma distribution [14,15], and the Gamma–Gamma distribution [16]. However, although these models comply with current data of irradiance fluctuations due to the joint effect of thermohaline gradient and air bubbles, neither of them have been evaluated for optical power fluctuations due exclusively to air bubbles. In this regard, the thermohaline gradient can mask the real impact of air bubbles on the perturbed wavefront at the receiver. Furthermore, oceanic turbulence due to the inhomogeneous thermohaline distribution represents a minor drawback compared with air bubbles or the absorption and scattering effect in deep waters, as the temperature and salinity of the underwater medium tend to stabilize at constant values [17]. However, the origin of air bubbles is highly heterogeneous (cavitation of vehicle propellers, phytoplankton photosynthesis, or zooplankton respiration [18,19]), and their presence can significantly distort the received footprint. Indeed, a few investigations have demonstrated that the performance degradation in UOWC links is due solely to air bubbles [20,21]. In [20], the dependence of the bit error rate (BER) of a UOWC link using different orders of pulse position modulation on bubble density and bubble size is experimentally analyzed in a water tank. In [21], closed-form expressions for the ergodic capacity and the BER were obtained over a composite channel model which considers the bubble-obstruction and turbulence effect. However, the proposed statistical model is purely mathematical and numerically obtained by using statistics of the generation, size, and horizontal movement of air bubbles. Unlike empirical measurements-based channel models which consider the complicated nature of real underwater environments, statistical channel models derived from theoretical simulations often require simplifying and assuming specific channel parameters. This can result in significant discrepancies between the simulated results and the actual behavior of underwater environments. Furthermore, it should be noted that the aforementioned works neglect the dispersive nature of the water, by ignoring different types of water in the performance analysis.

Recently, novel research has proposed a statistical UOWC channel model based on experimental measurements that considers the combined effect of the scintillation and obstruction of light due to air bubbles and the water turbidity [22]. Then, small air bubble-induced fluctuations are described by a generalized Gamma distribution, and large air bubble-induced fluctuations and light blockage are described by a mixture of two generalized Gamma distributions. Channel measurements suggest that the scattering effect reduces both the scintillation effect and the obstruction of light caused by air bubbles, by inducing a more resilient link at the expense of a higher pathloss value. However, the current research has ignored the scattering phenomenon because it makes use of statistical channel models that are not fitted to realistic underwater environments. In other words, the effectiveness of UOWC systems under air bubbles in turbid waters has not yet been investigated. Therefore, there remains a need for mathematical tools and expressions that can complete the UOWC system performance of submarine links when air bubbles of different sizes perturb the optical wavefront under different types of water.

In this paper, we analyze the UOWC system performance in terms of bit error rate (BER) and outage probability over underwater fading channels in the presence of small and large air bubbles and different levels of scattering following the mentioned empirical-based UOWC channel models [22]. Unlike [5,7,11], where the estimated PDF and the corresponding channel parameters are fitted when considering a joint effect of thermohaline gradient and air bubbles in tap water, the UOWC system is evaluated under UOWC statistical channel models where fitted PDFs and its parameters are based on measurements with optical power fluctuations due to uniquely from air bubbles of two different sizes and a wide range of in-suspension particle concentrations.

Although previous research has derived analytical expressions for BER using a generalized Gamma distribution, it is essential to note that these studies do not address the performance of optical systems under air bubble-induced fading and particle-induced scattering [23,24,25]. Furthermore, some of these papers utilize fitted parameters derived from numerical simulation, which do not accurately capture the complexity of the underwater environment. Consequently, their findings may have limitations when applied to optimizing and designing novel UOWC systems in realistic conditions, such as environments with air bubbles in turbid water. This work extends the knowledge of existing UOWC system performance based on empirical UOWC channel models because we analyze the impact of water turbidity, which considers the oceanic pathloss and the beamwidth expansion due to the scattering effect. In particular, we provide exact and asymptotic closed-form expressions for BER and outage probability over air bubble-induced fading channels. In fact, to our knowledge, there is no asymptotic analysis of BER and outage probability presented in the literature that can obtain novel insights into how channel model parameters affect the overall performance at high signal-to-noise (SNR) ratio. Therefore, by using the asymptotic behavior, a novel diversity order analysis for the different scattering scenarios is included. The presented results show that UOWC links under higher levels of scattering exhibit a lower average BER and outage probability than tap water scenarios. It follows, therefore, that particle-induced scattering can mitigate the random irradiance fluctuations and light blockages due to both small and large air bubbles. Monte Carlo simulations are included to verify the presented results.

The remainder of this article is organized as follows. In Section 2, the system and channel models adopted in this work are described for small and large air bubbles from experimental measurements. In Section 3, we derive new closed-form analytic and asymptotic expressions for the average BER and outage probability when adopting the proposed channel models. Numerical results and discussions are presented and analyzed in Section 4. Finally, we summarize the main conclusions and some future lines of this work in Section 5.

## 2. System and Channel Models

**Figure 1:**
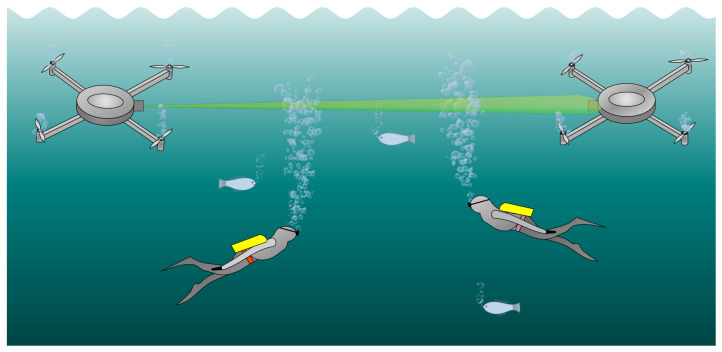
Schematic of a UOWC link application between two AUVs under air bubble scenario due to oxygen systems of scuba divers and AUV propellers.

Figure 1 illustrates a UOWC link between two autonomous underwater vehicles (AUV). In a practical underwater scenario, the transmitted beam is influenced by the underwater particles in terms of absorption and scattering, as well as air bubbles, which are generated by the cavitation effect of AUV propellers, marine flora and fauna respiration, and oxygen systems of scuba divers. A comprehensive model that contemplates the relation of these impairments in realistic scenarios is essential for accurately characterizing the UOWC channel and assessing the performance of UOWC systems. The following section presents the system and channel models adopted in this work, considering the environmental conditions prevalent in underwater settings from experimental measurements presented in [22].

### 2.1. System Model

Consider a single-input/single-output (SISO) UOWC system with an intensity modulation and direct detection (IM/DD) scheme due to the lower complexity and low cost with respect to coherent schemes [26]. The received electrical signal for the proposed UOWC system is given by

(1) $$y=L\cdot h_{b}\cdot R\cdot x+n,$$

where *L* is the pathloss due to the water turbidity and air bubbles, $h_{b}$ is the fading coefficient of the UOWC channel due to air bubbles, *R* is the detector responsivity, assumed hereinafter to be the unity, *x* is the transmitted optical power, and *n* is additive white Gaussian noise with zero mean and variance $N_{0}/2$. Hence, the received average electrical SNR in the absence of fading can be defined as $\gamma =P_{opt}^{2}T_{b}/N_{0}$, where $P_{opt}$ is the average transmitted optical power, and $T_{b}$ is the bit period.

### 2.2. Air Bubble-Induced Fading Model

Air bubble-induced fading is modeled using the generalized Gamma distribution and a mixture of the sum of two generalized Gamma distributions when considering small air bubbles and large air bubbles, respectively [22]. The generalized Gamma distribution is a versatile statistical model that includes the Gamma distribution, the Chi and Chi-squared distribution, the Exponential distribution, and the Weibull distribution, which are usually employed in modeling power fluctuations in radio-frequency (RF) and optical wireless systems [7,23,24]. In the context of underwater environments, the Exponential-Generalized Gamma mixture has been used to analyze the multihop UOWC systems performance under oceanic turbulence [25,27]. Furthermore, in [22], the generalized Gamma distribution and the mixture of the sum of two generalized Gamma distributions provide an excellent agreement for a wide range of water turbidity scenarios, as well as different air bubble populations with experimental measurements under controlled laboratory conditions [22]. It should be noted that the small and large terms are defined in relation to the beam width of the transmitted beam. When the diameter of an air bubble is comparable to or bigger than such a beam width, it can result in a partial or complete light blockage. In such instances, this constitutes a scenario of large air bubbles. Concerning the PDF, large air bubbles exhibit a bimodal behavior due to the combined effects of light attenuation caused by large bubbles and power fluctuations induced by particles and small bubbles formed through bubble collisions. Conversely, scenarios involving smaller bubbles are assumed to represent instances of small air bubbles, which induce power fluctuations around a given received mean power. All details regarding fitting the PDF to the experimental measurements can be found in [22].

The corresponding PDF for small air bubble-induced fading is given by ([22], Equation (7)).

(2) $$f_{h_{\mathrm{small}}}\left(h;a,d,p\right)=\frac{p}{a^{d}\Gamma \left(d/p\right)}\;h^{d-1}\;e^{-{\left(h/a\right)}^{p}},$$

where *h* is the random variable, Γ(·) is the Gamma function, *a* is a positive scale parameter, and *d* and *p* are positive shape parameters [28]. In the same way, the considered PDF of large air bubble-induced fading is given by ([22], Equation (8))

(3) $$f_{h_{\mathrm{large}}}\left(h;a_{1},d_{1},p_{1},a_{2},d_{2},p_{2},w\right)=w\cdot f_{h_{\mathrm{small}}}\left(h;a_{1},d_{1},p_{1}\right)+\left(1-w\right)\cdot f_{h_{\mathrm{small}}}\left(h;a_{2},d_{2},p_{2}\right),$$

where *w* is the proportion between the blockage effect, the first term, and the power fluctuation effect, the second term, such that w∈[0,1].

By fitting $f_{h_{\mathrm{small}}}\left(h\right)$ and $f_{h_{\mathrm{large}}}\left(h\right)$ channel models to experimental UOWC measurements, the specific values of the distribution parameters, as well as some parameters related to the UOWC channel such as the experimental propagation losses, $L_{T}$, the extinction coefficient of the UOWC link, *c*, the scintillation index, $\sigma^{2}$, and the coefficient of determination, $R^{2}$, are obtained with a 520 nm laser diode at a link distance of 3 m. For detailed information on the experimental equipment and the setup used in the measurements, including all relevant parameters and configurations, please refer to the comprehensive description provided in [22]. A summary of the parameters of each distribution and the remaining parameters derived from the measurement of the optical power fluctuations for different levels of commercial antacid is presented in Table 1 and Table 2 when considering small and large air bubble scenarios, respectively. As reported, both proposed models accurately fit the random behavior of the received optical power for both small and large air bubbles, with a coefficient of determination $R^{2}$ exceeding 0.95 for all evaluated scattering conditions.

## 3. Performance Analysis of UOWC Links under Air Bubble-Induced Fading

In this section, the performance of a SISO UOWC system under the proposed underwater channel in the presence of air bubbles and different water turbidity levels is analyzed using the average BER and the outage probability. Moreover, asymptotic expressions are obtained to shed light on the impact of the channel parameters on the system performance by revealing the diversity order and the coding gain.

### 3.1. Bit Error Rate Analysis

As a case study, we analyze the BER performance of a SISO UOWC system when adopting On-Off Keying (OOK) signaling for the case of perfectly known channel state information at the receiver. As stated in [29], the conditional average BER at the receiver for the case of equally likely transmitted symbols is given by

(4) $$P_{b}\left(E|h\right)=Q\left(\sqrt{2\gamma }\cdot L\cdot h_{b}\right),$$

where Q(·) represents the Gaussian-*Q* function. Hence, $P_{b}$ in the small air bubble scenario is obtained by averaging over the PDF of small air bubbles defined in Equation (2) as follows:

(5) $$P_{b_{\mathrm{small}}}=\int_{0}^{\infty }Q\left(\sqrt{2\gamma }\cdot L\cdot h_{b}\right)\cdot f_{h_{\mathrm{small}}}\left(h_{b}\right)\;dh_{b}.$$

By substituting Equation (2) into Equation (5), and making use of ([30], Equation (07.34.21.0012.01)), the exact closed-form expression for the average BER in the presence of small air bubbles can be obtained as follows

(6) $$P_{b_{\mathrm{small}}}\left(L,a,d,p\right)=\frac{p}{4\sqrt{\pi }{\left(L\;a\right)}^{d}\Gamma \left(\frac{d}{p}\right)L^{d}}\gamma^{-\frac{d}{2}}H_{2,2}^{1,2}\left[\frac{1}{{\left(L\;a\right)}^{p}}\gamma^{-\frac{p}{2}}|\begin{array}{c} \left(1-\frac{d}{2},\frac{p}{2}\right),\left(\frac{1-d}{2},\frac{p}{2}\right)\\ \left(0,1\right),\left(-\frac{d}{2},\frac{p}{2}\right), \end{array}\right],$$

where $H_{m,n}^{p,q}\left(\cdot \right)$ is the H-Fox function ([31], Equation (1.1.1)).

Similarly, the average BER when considering large air bubbles is obtained by averaging over the PDF of large air bubbles defined in Equation (3) as follows:

(7) $$P_{b_{\mathrm{large}}}=\int_{0}^{\infty }Q\left(\sqrt{2\gamma }\cdot L\cdot h_{b}\right)\cdot f_{h_{\mathrm{large}}}\left(h_{b}\right)\;dh_{b}.$$

From Equations (3) and (7) can be formulated as a weighted sum of integrals as follows:

(8) $$P_{b_{\mathrm{large}}}=\int_{0}^{\infty }Q\left(\sqrt{2\gamma }\cdot L\cdot h_{b}\right)\cdot w\cdot f_{h_{\mathrm{small}}}\left(h_{b};a_{1},d_{1},p_{1}\right)\;dh_{b}+\int_{0}^{\infty }Q\left(\sqrt{2\gamma }\cdot L\cdot h_{b}\right)\cdot \left(1-w\right)\cdot f_{h_{\mathrm{small}}}\left(h_{b};a_{2},d_{2},p_{2}\right)\;dh_{b}.$$

Thus, $P_{b_{\mathrm{large}}}$ can be easily derived from Equation (5) as a weighted sum of $P_{b_{\mathrm{small}}}$ as follows:

(9) $$P_{b_{\mathrm{large}}}\left(L,a_{1},d_{1},p_{1},a_{2},d_{2},p_{2},w\right)=w\cdot P_{b_{\mathrm{small}}}\left(L,a_{1},d_{1},p_{1}\right)+\left(1-w\right)\cdot P_{b_{\mathrm{small}}}\left(L,a_{2},d_{2},p_{2}\right).$$

Since the obtained exact closed-form expressions could obscure the impact of PDF channel parameters on the UOWC system performance, more mathematically tractable expressions are obtained based on the asymptotic behavior at high SNR. According to ([32], Proposition 1), the asymptotic BER at high SNR can be derived with the behavior of the PDF of the considered random variable ($h_{b}$) near the origin. Hence, the asymptotic BER at high SNR tends to $P_{b}≐{\left(G_{c}\gamma \right)}^{-G_{d}}$, where $G_{c}$ is the coding gain, $G_{d}$ is the diversity order, and ≐ is the asymptotic equality sign [32]. On a log–log scale $G_{c}$ and $G_{d}$ specify a relative horizontal shift and the slope of the average BER and outage curves in the asymptotic regime, respectively. Both PDFs expressed in Equations (2) and (3) can be approximated by a single polynomial term for $h_{b}\to 0$, i.e., near the origin, as follows

(10) $$f_{h_{\mathrm{small}}}\left(h_{b}\right)≐\frac{p}{a^{d}\Gamma \left(d/p\right)}\;h_{b}^{d-1},$$

and

(11) $$f_{h_{\mathrm{large}}}\left(h_{b}\right)≐w\frac{p_{1}}{a_{1}^{d_{1}}\Gamma \left(d_{1}/p_{1}\right)}\;h_{b}^{d_{1}-1}+\left(1-w\right)\frac{p_{2}}{a_{2}^{d_{2}}\Gamma \left(d_{2}/p_{2}\right)}\;h_{b}^{d_{2}-1}.$$

Then, by substituting Equation (10) into Equation (5), and making use of ([30], Equation (07.34.21.0009.01)), the asymptotic closed-form expression for the average BER in the presence of small air bubbles can be solved as follows:

(12) $$P_{b_{\mathrm{small}}}≐\frac{p\;\Gamma \left(\frac{1+d}{2}\right)}{2\;\sqrt{\pi }\;d{\left(L\;a\right)}^{d}\;\Gamma \left(\frac{d}{p}\right)}\gamma^{-\frac{d}{2}}.$$

Since the $P_{b_{\mathrm{large}}}$ is a weighted sum of $P_{b_{\mathrm{small}}}$, the asymptotic closed-form expression for the average BER can be solved as follows:

(13) $$P_{b_{\mathrm{large}}}≐\frac{w}{2\sqrt{\pi }}\frac{p_{1}\Gamma \left(\frac{1+d_{1}}{2}\right)}{{\left(La_{1}\right)}^{d_{1}}d_{1}\Gamma \left(\frac{d_{1}}{p_{1}}\right)}\gamma^{-\frac{d_{1}}{2}}+\frac{1-w}{2\sqrt{\pi }}\frac{p_{2}\Gamma \left(\frac{1+d_{2}}{2}\right)}{{\left(La_{2}\right)}^{d_{2}}d_{2}\Gamma \left(\frac{d_{2}}{p_{2}}\right)}\gamma^{-\frac{d_{2}}{2}}.$$

### 3.2. Outage Probability Analysis

An outage probability analysis for a SISO UOWC system under the UOWC channel model in the presence of air bubbles proposed in [22] is presented here to assess the completeness of the UOWC system performance. The outage probability is defined as the instantaneous SNR falls below a predefined threshold, $\gamma_{th}$ as [29]

(14) $$\mathrm{OP}=P(\gamma_{T}\leq \gamma_{th}),$$

where $\gamma_{T}=4\cdot \gamma \cdot {\left(h_{b}\cdot L\right)}^{2}.$ Therefore, Equation (14) can be expressed as

(15) $$\mathrm{OP}=P\left(4\cdot \gamma \cdot {\left(h_{b}\cdot L\right)}^{2}\leq \gamma_{th}\right)=\int_{0}^{\sqrt{\gamma_{th}/4L^{2}\gamma }}f_{h_{b}}\left(h\right)dh=F_{h_{b}}\left(\sqrt{\frac{1}{4L^{2}\bar{\gamma }}}\right),$$

where $\bar{\gamma }=\frac{\gamma }{\gamma_{th}}$ is the normalized SNR, and $F_{h_{b}}\left(\cdot \right)$ is the cumulative distribution function (CDF). The definition of the normalized SNR allows for the extrapolation of outage probability results to any predefined threshold value. The corresponding CDF when considering small air bubbles is obtained as follows ([28], Equation (2)).

(16) $$F_{h_{\mathrm{small}}}\left(h;a,d,p\right)=\frac{\nu (\frac{d}{p},{\left(h/a\right)}^{p})}{\Gamma \left(\frac{d}{p}\right)},$$

where ν(·,·) is the lower incomplete Gamma function [33]. Hence, by substituting Equation (16) into Equation (2.3) of [33], $F_{h_{\mathrm{small}}}\left(\cdot \right)$ can be easily derived, as follows:

(17) $$F_{h_{\mathrm{small}}}\left(h;a,d,p\right)=1-\frac{\Gamma (\frac{d}{p},{\left(h/a\right)}^{p})}{\Gamma \left(\frac{d}{p}\right)},$$

where Γ(·,·) is the upper incomplete Gamma function [33]. Therefore, the corresponding outage probability when small air bubbles are considered can be easily derived by substituting Equation (17) into (15), as follows:

(18) $${\mathrm{OP}}_{\mathrm{small}}=1-\frac{\Gamma \left(\frac{d}{p},\frac{1}{{\left(aL\right)}^{p}}{\bar{\gamma }}^{-\frac{p}{2}}\right)}{\Gamma \left(\frac{d}{p}\right)}.$$

In the same way that the average BER, the outage probability at high SNR also tends to $\mathrm{OP}≐{\left(O_{c}\gamma \right)}^{-O_{d}}$, where $O_{c}$ is the coding gain, and $O_{d}$ is the diversity order. Hence, by replacing Equation (10) into Equation (15), we obtain the asymptotic behavior of the outage probability at high SNR for small air bubbles as follows:

(19) $${\mathrm{OP}}_{\mathrm{small}}≐\frac{p}{{\left(aL\right)}^{d}d\;\Gamma \left(\frac{d}{p}\right)}{\bar{\gamma }}^{-\frac{d}{2}}.$$

Based on Equation (3), the CDF of large air bubble-induced fading can be expressed as

(20) $$F_{h_{\mathrm{large}}}\left(h;a_{1},d_{1},p_{1},a_{2},d_{2},p_{2},w\right)=w\cdot F_{h_{\mathrm{small}}}\left(h;a_{1},d_{1},p_{1}\right)+\left(1-w\right)F_{h_{\mathrm{small}}}\left(h;a_{2},d_{2},p_{2}\right).$$

Hence, the outage probability in the presence of large air bubbles is derived by substituting Equation (17) into Equation (20) as follows:

(21) $${\mathrm{OP}}_{\mathrm{large}}=w\left(1-\frac{\Gamma \left(\frac{d_{1}}{p_{1}},\frac{1}{{\left(a_{1}L\right)}^{p_{1}}}{\bar{\gamma }}^{-\frac{p_{1}}{2}}\right)}{\Gamma \left(\frac{d_{1}}{p_{1}}\right)}\right)+\left(1-w\right)\left(1-\frac{\Gamma \left(\frac{d_{2}}{p_{2}},\frac{1}{{\left(a_{2}L\right)}^{p_{2}}}{\bar{\gamma }}^{-\frac{p_{2}}{2}}\right)}{\Gamma \left(\frac{d_{2}}{p_{2}}\right)}\right).$$

Finally, by applying Equation (11) into Equation (15), we obtain the asymptotic of the outage probability at high SNR for large air bubbles as follows:

(22) $$OP_{\;\mathrm{large}}≐w\frac{{\left(\frac{1}{a_{1}L}\right)}^{d_{1}}}{\Gamma \left(\frac{d_{1}+p_{1}}{p_{1}}\right)}{\bar{\gamma }}^{-\frac{d_{1}}{2}}+\left(1-w\right)\frac{{\left(\frac{1}{a_{2}L}\right)}^{d_{2}}}{\Gamma \left(\frac{d_{2}+p_{2}}{p_{2}}\right)}{\bar{\gamma }}^{-\frac{d_{2}}{2}}.$$

### 3.3. Diversity Order Analysis

As mentioned before, the average BER and the outage probability tend to $P_{b}≐{\left(G_{c}\gamma \right)}^{-G_{d}}$ and $\mathrm{OP}≐{\left(O_{c}\bar{\gamma }\right)}^{-O_{d}}$. As stated in [32], the BER and outage performance exhibit identical diversity orders for sufficiently large SNR, hence $G_{d}=O_{d}$. The resulting diversity order is useful for evaluating in a unifying manner and building insights about the impact of the underwater channel parameters on the UOWC system performance. From Equations (12) and (19), it can be shown that the diversity order, $G_{d}$, when considering small air bubbles is given by

(23) $$G_{d_{small}}=\frac{d}{2}.$$

In the case of large bubbles, both the average BER and outage probability tend to $P_{b}≐{\left(G_{c_{1}}\gamma \right)}^{-G_{d_{1}}}+{\left(G_{c_{2}}\gamma \right)}^{-G_{d_{2}}}$ and $\mathrm{OP}≐{\left(O_{c_{1}}\bar{\gamma }\right)}^{-O_{d_{1}}}+{\left(O_{c_{2}}\bar{\gamma }\right)}^{-O_{d_{2}}}$, where $G_{d_{i}}=O_{d_{i}}$. Therefore, the diversity order refers to the exponent of γ and $\bar{\gamma }$ in Equations (13) and (22), which determine the slope of the average BER and outage probability at high SNR, respectively. Mathematically, the smallest exponent dominates the behavior of these expressions. Thus, the diversity order can be expressed as the minimum of $G_{d_{1}}$ and $G_{d_{2}}$. Consequently, the diversity order can be obtained from Equations (13) and (22) as follows:

(24) $$G_{d_{large}}=\frac{\min(d_{1},d_{2})}{2}.$$

## 4. Numerical Results and Discussion

In this section, the average BER and outage probability of UOWC links under air bubble-induced scattering with different levels of water turbidity are evaluated. Furthermore, we provide Monte Carlo simulation results to verify the proposed analytical and asymptotic closed-form expressions.

Regarding the numerical simulation, a quasi-analytical Monte Carlo simulation approach with an equivalent noise source to improve computational efficiency has been implemented. Due to the long simulation time involved, simulation results only up to $10^{-9}$ are included in this manuscript. Therefore, the number of bits used in our simulations is $10^{10}$ to ensure precision [34]. As mentioned above, OOK modulation is employed with a rectangular pulse shape. The output waveform takes predefined values corresponding to a 1 or 0, with each bit occurring with a probability of 0.5. It should be noted that no channel coding scheme was incorporated. The transmitted signal is detected using a matched filter at the receiver, with an impulse response matching the rectangular pulse shape. This filter maximizes the sampled SNR, and a maximum likelihood (ML) detector minimizes symbol error probability. Simulations are conducted in baseband, without considering carrier frequency and frequency band.

Concerning the empirical-based channel model, it must be noted that different channel conditions and fitted parameters are obtained from empirical measurements at a link distance of 3 m and a wavelength of 520 nm, which are summarized in Table 1 and Table 2 [22]. The measurement-based channel model allows for a more accurate estimation of the average BER and outage probability, accurately reflecting the complex relationships among absorption, particle-induced scattering, and air bubbles. Additionally, different levels of absorption and scattering were selected to represent a wide range of water types. For instance, for 0 mg/L of antacid, the extinction coefficient is $c=0.16\;m^{-1}$, which can be compared to the extinction coefficient of clear ocean water or the Jerlov IB water type [3,35]. In the case of the most turbid scenario, for 14.5 mg/L of antacid, $c=1.24\;m^{-1}$, the corresponding extinction coefficient is near the Jerlov 5C water type [35].

### 4.1. Diversity Order Performance

**Figure 2:**
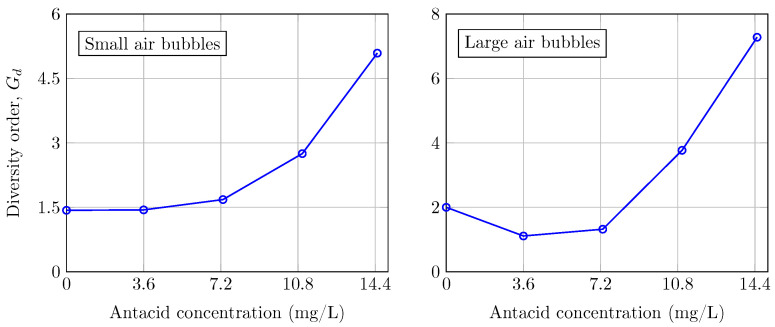
Diversity order when considering small air bubbles and large air bubbles for different levels of antacid in the experimental UOWC link.

In Figure 2, we show the diversity order of each level of water turbidity when considering small air bubbles and large air bubbles when considering the same UOWC system. Firstly, in the presence of small air bubbles, the diversity order exhibits an exponentially increasing trend as the concentration of antacid grows, demonstrating a proportional relationship between the two variables. For large air bubbles, we observe that at the lowest level of antacid, i.e., 3.6 mg/L, the diversity order decreases slightly compared with tap water, i.e., 0 mg/L. It may be attributed to the unique and singular impact of partial and total light blockages caused by large bubbles. This effect diminishes at higher antacid concentrations with a more significant collection of scattered photons, as detailed in [22]. For this reason, after the initial singularity in the diversity order of the large air bubble scenario, both graphs provide a coherent and valuable insight into the exponential growth of diversity order in relation to water turbidity.

### 4.2. Small Air Bubble Scenario

**Figure 3:**
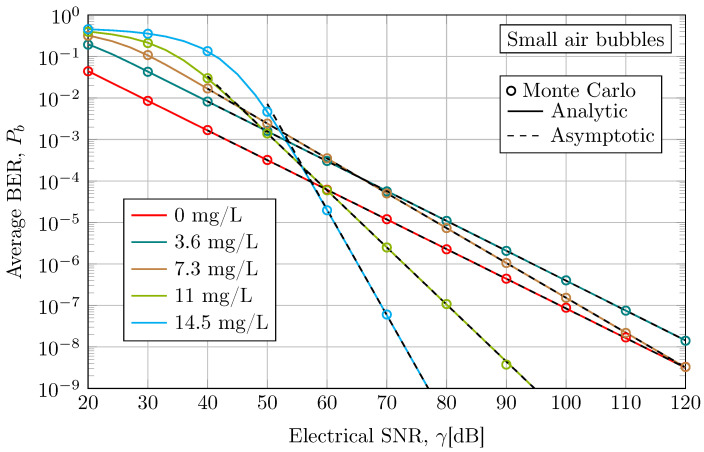
Average BER performance when considering an empirical UOWC channel in the presence of small air bubble-induced fading under different levels of antacid concentration at a link distance of 3 m and a wavelength of 520 nm.

In Figure 3, the average BER of a SISO UOWC system under small air bubbles for different levels of scattering are compared. As can be readily observed, the analytical and asymptotic results show an excellent agreement with Monte Carlo simulation results for all considered cases by validating the accuracy of the exact and asymptotic closed-form expressions in Equations (6) and (12), respectively. Firstly, it is interesting to note that for scenarios with a low antacid concentration, the asymptotic expression approaches Monte Carlo and analytic results even at low SNR faster than higher antacid concentrations. Secondly, as one would expect, the high pathloss due to increased antacid concentration deteriorates the average BER performance compared with the tap water case, as evident in the 3.6 mg/L scenario, which shows a higher average BER than the 0 mg/L scenario. However, the increase in diversity order due to higher water turbidity, as shown in Figure 2, reverses this trend at high SNR regime, especially for more turbid scenarios. While the cases with 3.6 mg/L and 7.3 mg/L of antacid exhibit higher average BER than the 0 mg/L case within the presented SNR range, the scenarios with the highest water turbidity, i.e., 11 mg/L and 14.5 mg/L of antacid concentration, significantly improve the performance of the UOWC system at high SNR under the same population of air bubbles. Thus, antacid-induced scattering mitigates the impact of air bubbles on the performance of UOWC systems. For instance, at 40 dB, the average BER for 14.5 mg/L is $1.35\times 10^{-1}$, while for 0 mg/L, it is $1.7\times 10^{-3}$. However, at 70 dB, the average BER for 14.5 mg/L is $6\times 10^{-6}$, while for 0 mg/L, it is $1.2\times 10^{-5}$. In practice, when the system is assured of having an SNR greater than 58 dB, the scenario with the highest turbidity will exhibit better performance in terms of average BER compared with the scenario with tap water. To illustrate this point, let us consider the natural optical beam spreading and geometric losses due to scattering. Hence, this effect can offer a great degree of robustness to air bubble-induced scattering and light blocking. These findings are in line with previous simulation and experimental results reported in [12,22], where the impact of pointing error and scintillation index also decreases as the water gets more turbid, respectively. Therefore, we conclude that this change in trend can be perfectly attributable to an increase in the severity of scattering. However, this interpretation is limited to the 3 m experimental water tank from which fitted channel model parameters have been obtained. The impact of scattering over longer link distances may not be accurately reflected. For shorter distances, the attenuation effect of turbid waters might be less significant than the beam spreading effect, potentially improving BER performance, as mentioned above. Nevertheless, over longer distances, the pathloss calculated from the Beer–Lambert law as $e^{-cd}$, where *d* is the link distance, becomes significantly greater in turbid waters, potentially resulting in higher BER performance. Therefore, beam spreading due to scattering can benefit short-range communication links by mitigating air bubble blockage, but overall performance can degrade over longer link distances due to increased pathloss.

**Figure 4:**
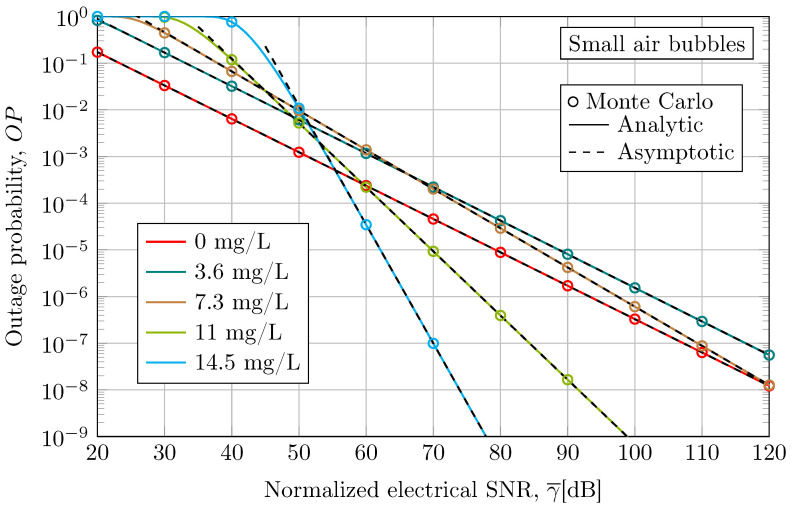
Outage performance when considering an empirical UOWC channel in the presence of small air bubble-induced fading under different levels of antacid concentration at a link distance of 3 m and a wavelength of 520 nm.

The outage probability for small air bubbles is represented in Figure 4. As expected, the Monte Carlo simulation results agree with the analytical and asymptotic expression described in Equations (18) and (19), respectively. Note that the outage probability results reveal similar insights as the average BER into the impact of water turbidity on UOWC system performance.

### 4.3. Large Air Bubble Scenario

**Figure 5:**
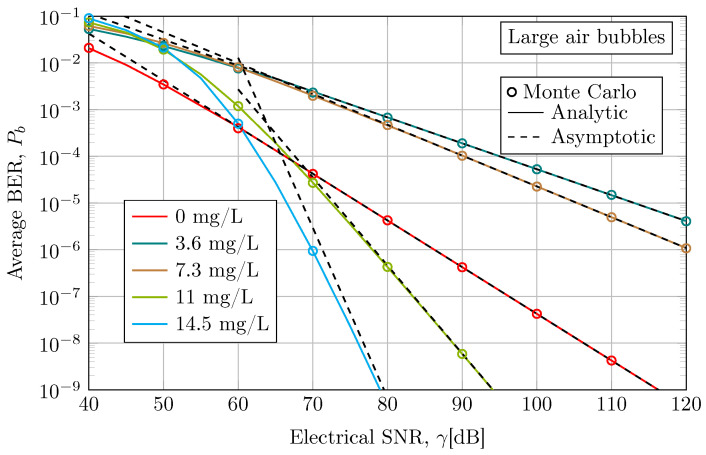
Average BER performance when considering an empirical UOWC channel in the presence of large air bubble-induced fading under different levels of antacid concentration at a link distance of 3 m and a wavelength of 520 nm.

In Figure 5, we consider the average BER in the presence of large air bubble-induced fading for different water turbidity levels. As can be observed, the considered antacid concentration levels are similar to the small air bubble scenarios. Both analytic and asymptotic results demonstrate behavior similar to Monte Carlo simulation results, indicating the accuracy of the exact and asymptotic closed-form expressions obtained in Equations (9) and (13), respectively. In addition, Monte Carlo and analytic results support the convergence of the asymptotic solution, which is again faster for less turbid water cases. It can be observed that the asymptotic behavior of the scenario with a concentration of 14 mg/L of antacid does not fit as fast and accurately as the rest of the scattering scenarios. This is because this scenario exhibits a higher diversity order, i.e., a greater slope in the BER curve. Nonetheless, asymptotic results can be used as a tight upper bound across the presented SNR range.

As discussed in Section 4.2, the behavior of the average BER can be categorized into two regions. Before 60 dB, the average BER is lower when considering a tap water scenario, where the scattering effect is insignificant. However, after 60 dB, low average BER results are achieved in most turbid water scenarios. At 40 dB, the average BER for 14.5 mg/L is $9.1\times 10^{-2}$, while for 0 mg/L, it is $2.1\times 10^{-2}$. However, at 70 dB, the average BER for 14.5 mg/L is $9.4\times 10^{-7}$, while for 0 mg/L, it is $4.2\times 10^{-5}$. Although the scenarios with 3.6 mg/L and 7.3 mg/L of antacid show a higher average BER than the tap water scenario in all the evaluated SNR ranges, after 60 dB, the 7.3 mg/L scenario shows a lower average BER than the 3.6 mg/L scenario. This should be attributable to an increase in the absorption coefficient, i.e., a higher pathloss, in 3.6 mg/L and 7.3 mg/L scenarios with respect to the clear water case. However, it seems that the scattering coefficient remains constant for both cases.

**Figure 6:**
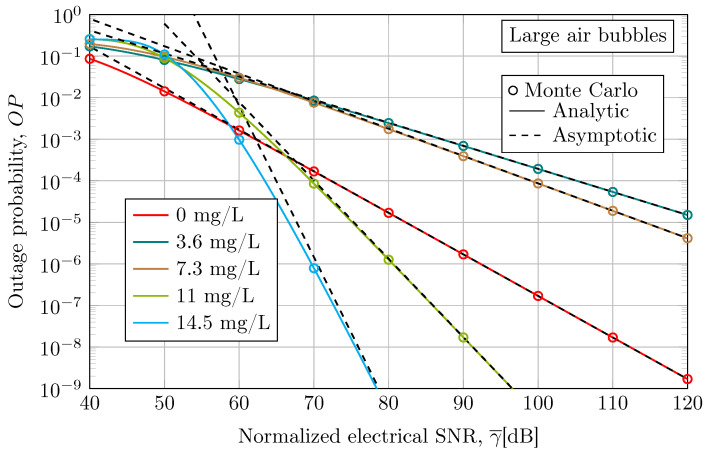
Outage performance when considering an empirical UOWC channel in the presence of large air bubble-induced fading under different levels of antacid concentration at a link distance of 3 m and a wavelength of 520 nm.

In Figure 6, the outage performance results of the UOWC system under large air bubbles for different levels of water turbidity are plotted. Monte Carlo simulations results validate the analytic and asymptotic results obtained from Equations (21) and (22). The outage probability confirms the conclusions obtained from the above-average BER performance.

## 5. Conclusions

In this paper, novel closed-form expressions have been developed in order to compute the average BER and outage probability of UOWC systems under empirical underwater channels in the presence of small and large air bubbles when different scattering levels are contemplated. Furthermore, simple asymptotic expressions are also obtained, which allows us to analyze the diversity order of the UOWC system of each scenario. Monte Carlo simulations verify the obtained analytic and asymptotic results.

For the first time, the proposed system performance contemplates an empirical underwater optical channel model obtained from experimental data, which considers the impact of scattering in terms of pathloss and beam spreading. The considered UOWC measurement-based channel model is able to capture the inherent complexities and variability of absorption, scattering, and power fluctuations due to air bubbles with greater accuracy than theoretical models based on simulations. Hence, the accuracy of the empirical modeling improves the average BER and outage estimation under realistic environments. In particular, our results prove that the relative impact of air bubbles on BER performance becomes less significant as water turbidity increases at short link distances. The reason behind this is that the diversity order highly depends on the scattering effect, increasing for higher antacid concentrations, i.e., more turbid waters. Hence, our performance analysis provides novel insights into the existing UOWC systems in realistic water bodies, since the proposed fading statistical models in [22] consider the air bubble-induced fading and light blockage, as well as the water turbidity and the scattering effect.

Since the performance analysis of UOWC systems under a realistic oceanic medium is a critical investigation in current UOWC networks, our technical contribution is particularly important to the engineers working on UOWC systems optimization and design. Moreover, the use of experimental channel models allows for the validation and adjustment of theoretical models, thereby improving their robustness and practical applicability. Some interesting extensions of our work, including the performance evaluation of more sophisticated schemes such as coherent modulation for UOWC systems, as well as Multiple-Input/Multiple-Output (MIMO) UOWC systems, will be considered as the subject of our future research. Furthermore, the presented UOWC channel performance could be enhanced by exploring the impact of scattering and attenuation over extended link distances to provide a more comprehensive understanding of UOWC system performance.

## Figures and Tables

**Table 1:** Experiment results of fitting parameters for generalized Gamma, total losses, the attenuation coefficient, and the scintillation index under small air bubble scenario for different turbidity levels.

Antacid [mg/L]	(a,d,p)	$L_{T}$ [dB]	c [$m^{-1}$]	$\sigma^{2}$	$R^{2}$
0	(1.73, 1.43, 7.95)	6.8	0.16	0.22	0.99
3.6	(1.72, 1.44, 6.28)	11.7	0.54	0.23	0.99
7.3	(1.59, 1.68, 4)	14.7	0.77	0.19	0.98
11	(1.19, 2.75, 3.05)	17.4	0.98	0.14	0.99
14.5	(0.79, 5.09, 2.41)	20.9	1.24	0.09	0.98

**Table 2:** Experiment results of fitting parameters for generalized Gamma, total losses, the attenuation coefficient, and the scintillation index under large air bubble scenario for different turbidity levels.

Antacid [mg/L]	$\left(a_{1},d_{1},p_{1}\right)$	$\left(a_{2},d_{2},p_{2}\right)$	*w*	$L_{T}$ [dB]	c [$m^{-1}$]	$\sigma^{2}$	$R^{2}$
0	(0.028, 2, 1.2)	(1.38, 29.38, 22.7)	0.2	5.0	0.16	0.32	0.95
3.6	(0.05, 1.110, 0.85)	(1.38, 13.95, 12.8)	0.23	9.9	0.54	0.30	0.98
7.3	(0.06, 1.320, 0.85)	(1.39, 11.93, 11.7)	0.23	12.9	0.77	0.29	0.98
11	(0.047, 3.77, 1.060)	(1.36, 10.87, 10.55)	0.26	15.6	0.98	0.27	0.98
14.5	(0.037, 7.28, 0.98)	(1.33, 10.87, 10.2)	0.25	19.1	1.24	0.21	0.98

## Data Availability

Data will be made available on request.

## Abbreviations

The following abbreviations are used in this manuscript:

AUV	Autonomous Underwater Vehicles
BER	Bit Error Rate
CDF	Cumulative Distribution Function
IM/DD	Intensity Modulation/Direct Detection
OOK	On-Off Keying
PDF	Probability Density Function
SISO	Single-Input/Single-Output
SNR	Signal-to-Noise Ratio
UOWC	Underwater Optical Wireless Communications
